# Antisocial punishment in two social dilemmas

**DOI:** 10.3389/fnbeh.2015.00107

**Published:** 2015-04-29

**Authors:** Enrique Fatas, Guillermo Mateu

**Affiliations:** ^1^School of Economics, University of East AngliaNorwich, UK; ^2^Laboratory for Social Sciences and Behavioral Analysis, Finance, Control, and Law Department, Burgundy School of BusinessDijon, France

**Keywords:** anti social behavior, punishment, public goods, coordination, experiments, C92

## Abstract

The effect of sanctions on cooperation depends on social and cultural norms. While free riding is kept at bay by altruistic punishment in certain cultures, antisocial punishment carried out by free riders pushes back cooperation in others. In this paper we analyze sanctions in both a standard public goods game with a linear production function and an otherwise identical social dilemma in which the minimum contribution determines the group outcome. Experiments were run in a culture with traditionally high antisocial punishment (Southern Europe). We replicate the detrimental effect of antisocial sanctions on cooperation in the linear case. However, we find that punishment is still widely effective when actions are complementary: the provision of the public good significantly and substantially increases with sanctions, participants punish significantly less and sanctions radically transform conditional cooperation patterns to generate significant welfare gains.

## Introduction

Social dilemmas have been extensively analyzed in the lab when the team's output is a linear function of the joint contribution to the team (in the so-called voluntary contribution mechanism, VCM). The provision of the public good typically declines over time in the vast majority of studies. However, certain mechanisms, such us communication, the inclusion of a threshold and sanctions, make the provision of the public good to be stable or even increasing over time (see Chaudury, 2011, for a comprehensive survey). This study contributes to the behavioral analysis of punishment, testing its effectiveness in two different social dilemmas.

The net effect of costly sanctions on the linear provision of public goods is a non-trivial issue. While evidence strongly suggests punishment may boost contributions to the public good, cooperation gains do not always compensate the cost of punishment. Moreover, its effectiveness seems to be heavily mediated by cultural effects; Herrmann et al. (2008) is a very well-known cross cultural study of how anti-social punishment may decrease the provision of public goods in societies governed by weak social norms. The behavioral characterization of sanctions is not particularly challenging in the VCM. Low-contributors free ride on the decisions of others and do not generate collective benefits. So, when cooperators punish free riders, sanctions are intuitively labeled as *social*. When free riders punish cooperators, their behavior is labeled as *anti-social*, because it does not serve the group, but individual objectives like revenge. Herrmann et al. (2008) rely on this characterization. Antisocial punishment may be used for several reasons, and others authors have defined antisocial punishment in a slightly different way. Bochet et al. (2006) and Cinyabuguma et al. (2006) use the term perverse punishment when the punisher contributes less than the others. In our design, we follow Herrmann et al. (2008) arguing that retaliation here can only be blind since each participant is never informed of the punishment that each individual assigned to him.

In many real life situations, public goods do not have an aggregative production function. The weakest link game mechanism (WLM) depicts the extreme case in which the minimum effort level determines the team's output, and has been widely used to explore coordination problems in organizations (Brandts et al., 2015). In this paper we extend the analysis of social sanctions to the WLM. As in Croson et al. (2005), we consider two social dilemma games, identically framed. Participants in the experiment repeatedly interact with the same players, first without punishment, then with the possibility of sanctioning each other at a cost (as in Herrmann et al., 2008).

In both environments participants make a simple and identically framed allocation decision. While in the VCM provision of the public good follows the sum of allocations, in the WLM it depends on the minimum allocation in the group. The only difference between both protocols is one line in the instructions describing the production function as a function of the average (minimum) individual allocation to the public account in the VCM (WLM). Our main objective is to analyze the effectiveness of punishment in this non-linear environment. How does this change in the production function may affect sanctioning patterns? Our rationale follows two simple but powerful intuitions. The first one relates to the lack of a clear-cut theoretical prediction for the behavioral logic of social antisocial sanctions in the WLM. The second, with the existence of culture effects described above. We describe these two intuitions one by one.

Even when individuals make very similar (and non-trivial) allocation decisions in the two environments, the logic of sanctions is fundamentally altered. While social punishment (sanctioning low contributors) may pursue similar objectives in both games (to increase cooperation of all, and expand the group welfare), the behavioral rationale for low contributions is quite different. While in the VCM low contributors free ride on the contributions of others (and drive the group away from the social optimum of full provision), in the WLM low contributors do not benefit from the contribution of others, as only the minimum contribution, their low contribution, determines the group outcome (and, again, drives the group away from the social optimum). As every single contribution profile is an equilibrium of the stage game in the WLM, low contributors' actions may be the consequence of pessimistic beliefs in a sophisticated equilibrium selection problem, rather than the outcome of selfish intentions (e.g., free riding on others). If the actions of low contributors are judged on the basis of their consequences on the group outcome, social punishment could be as prevalent (and cooperation enhancing) as in the VCM. As long as their decisions are considered to be, at least partially, the consequence of mistakes, wrong beliefs, or aversion to the strategic uncertainty of the game, we could expect social punishment to be less intense than in the VCM, at the cost of less public good provision.

Note that the analysis of antisocial punishment leaves a similarly open question. Antisocial punishment may follow very different logics depending on the information individuals receive about punishers in the previous round. From blind retaliation of the rest of the group, to targeted retaliation of those who punished, or the perverse punishment of those who contributed or punished less than the average, or punished contributors, the behavioral analysis of antisocial punishment has identified very different rationales for antisocial behavior (see Bochet et al., 2006; Cinyabuguma et al., 2006; Denant-Boemont et al., 2007; Nikiforiakis, 2008 for a discussion).

As we are interested in sanctions as cooperation enhancement devices, we do not provide individuals any information about punishment decisions in the previous round. So, in our setting, antisocial punishment cannot be related to targeted retaliation, as in Nikiforiakis (2008), or an attempt to sanction those who do not punish enough, or punish the wrong target (see Denant-Boemont et al. (2007) for a discussion of how sanctions my be driven by a desire to punish others on the basis of their sanctions to third parties). In our experiment, participants do not receive information about the distribution of sanctions chosen by the other participants, and their correspondence with the vector of contributions to the public good^1^.

Still, the differences between both environments are important. While the existence of antisocial punishment in the VCM has been (negatively) linked to the existence of survey based social norms outside the laboratory (related to civic cooperation and the rule of law), in the WLM the effect and interpretation of social norms becomes more problematic. As Herrmann et al. (2008) make it clear, “Social norms [… ] refer to widely shared views about acceptable behaviors and the deviations subject to possible punishment” (see p. 1365). As discussed above, social punishment in both games share at least the desirability of cooperation, for the sake of the group welfare. The extent to which antisocial punishment is perceived as acceptable is one of the open questions this study tries to answer.

Antisocial punishment, defined as sanctions targeting co-operators^2^, cannot be driven by social welfare maximizing intentions in either the VCM or the WLM. Blind retaliation in both environments cannot be excluded either. However, the lowest contributor is in a very different position in the VCM and the WLM games. As discussed above, she may have contributed less for very different reasons: selfishness in the VCM vs. strategic risk aversion or over-pessimistic beliefs about the actions of others in the WLM. Moreover, because the lowest contributors fully determine the group outcome only in the WLM, they may become focal points, and be easily targeted by the rest of the group. If becoming focal targets helps them to quickly adjust upwards their contributions, antisocial punishment should be less frequent and less intense (at the cost of more focalized punishment).

If the intensity of social punishment they receive is perceived as particularly unfair because low contributors do not benefit from the contribution of others, blind retaliation could ignite an escalation of highly detrimental sanctions. As high co-operators pay a relatively higher price in the WLM (they do not benefit from their individual contributions as the public good is determined by the minimum allocation), this escalation of antisocial punishment could feedback “social” punishment motivated by retaliation. The net effect of both behavioral forces on antisocial punishment is impossible to predict without a controlled behavioral test like the one we run in this paper.

To address these issues we run a series of laboratory experiments in one particular culture (Southern Europe), where antisocial punishment has traditionally prevented sanctions to induce full cooperation in linear public goods. The rationale for this decision is not difficult to explain. If the virtuous circle based on the acceptance of (social) punishment of low contributors and the absence of retaliation based escalation holds, sanctions could yield a positive result even in Southern Europe. If the vicious circle of antisocial punishment driven by the lack of guilt in low cooperators (because they never profited from the actions of others) triggers a generalization of social and antisocial punishment, sanctions in the WLM could generate even less provision of the public good, particularly in a location with weak norms of civic cooperation.

Our results are clear. We start by replicating the finding that sanctions are of very limited use in some cultures in linear environments like the VCM. We then document that the production technology has significant and vast consequences in the effectiveness of sanctions. While antisocial punishment is still largely present in the VCM, social punishment dominates in the WLM. Individuals punish significantly less in the WLM than in the VCM, and punishment generates large and significant welfare gains. Antisocial punishment is rare in the WLM, and low contributors do not retaliate. Interestingly, cooperation adjustments are massively asymmetric in the WLM, as cooperators do not adjust down their contributions when contributing more than others, at a huge personal cost, while upward adjustments are large and significantly stronger than in the VCM.

The rest of the paper goes as follows. Next section reviews the literature. After this, the subsequent section presents the experimental design and procedures. The next section introduces the quantitative analysis of contributions, punishment and efficiency for the two games simultaneously. Finally, the last section concludes.

## Literature review

The effects of both social and antisocial sanctions in cooperation have largely been studied (from Fehr and Gächter, 2000, 2002, to Nowak, 2006, and Rand and Nowak, 2013). The analysis of sanction in other species includes the study of wasps (Tibbetts and Dale, 2004) and monkeys (Hauser, 1992). Punishment mechanisms in humans were analyzed first in common-pool resource games (Ostrom et al., 1992) and social dilemmas (Yamagishi, 1986). Fehr and Gächter (2000, 2002) document the effect of sanctions in linear public good games, and show how cooperation pays because defectors suffer the cost of being punished by cooperators (see also Fehr and Fischbacher, 2003; Almenberg et al., 2011). The consequence is that cooperation may co-evolve with punishment through group selection (Boyd et al., 2003; Eckel et al., 2014).

The seminal distinction between social and antisocial punishment starts with Herrmann et al. (2008) and their cross culture analysis of sanctions in linear social dilemmas. In their study, they categorize as social punishment sanctions coming from individuals within a group who contribute more than the subjects they punish. Low contributors do not only free ride on the contributions of cooperators but harm the group fitness, by not exploiting the positive group externality associated with contributions to the public good. Sanctions of low cooperators are labeled as social punishment because “from the perspective of the punisher the target member behaved less pro-socially than the punisher” (p. 1636). Sanctions of high cooperators are labeled as antisocial.

Interestingly, a non-negligible fraction of participants in experiments are willing to pay to punish cooperators (Shinada et al., 2004; Cinyabuguma et al., 2006; Denant-Boemont et al., 2007; Dreber et al., 2008; Nikiforiakis, 2008; Gächter and Herrmann, 2009; Wu et al., 2009; Ellingsen et al., 2013; Hauser et al., 2014). Herrmann et al. (2008) and Gächter et al. (2010) show how the intensity of antisocial punishment received by cooperators across different cultures critically determines the effectiveness of sanctions. Only when the frequency and intensity of antisocial punishment is moderate, as in the original studies by Fehr and Gächter (2000, 2002), run in Western Europe, sanctions effectively allow cooperators to increase their fitness in the long run through cooperation gains.

In this paper we explicitly consider the role of punishment in different social dilemmas. We specifically focus in the consistency of social and antisocial punishment across different versions of the same public goods game. A large number of papers have studied interesting variations of punishment designs, (e.g., Gintis, 2000; Carpenter and Matthews, 2002; Carpenter et al., 2004). Masclet et al. (2003) study the power of non-material social sanctions and find that they still increase contributions in the short run, but only material (monetary) sanctions are effective in the long term. Fudenberg and Pathak (2010) find that individuals are willing to incur in costly punishment even if sanctions are not observed until the end of the session, supporting the idea that they somehow enjoy getting engaged in punishment. Quervain et al. (2004) document how punishers' personal satisfaction activates the dorsal striatum.

To the best of our knowledge, our study pioneers in studying the effects of costly sanctions in two different public goods games. In both cases, subjects contribute to a collective account and get similar collective benefits. In addition, games are framed as an identical allocation problem. The only difference comes from the team output's technology. In the first one (the VCM) the collective account profits are determined by the linear aggregation of contributions (the average contribution to the team account), while in the second (the WLM) is a multiple of the minimum contribution.

The main strategic differences between our games are two. First, while a unique inefficient equilibrium exists in the VCM, every symmetric contribution profile is equilibrium of the stage game in the WLM. The intuition is that from every symmetric profile, unilateral deviations never pay. By contributing more individuals do not expand the public good, and still pay for the larger contribution. By contributing less, players reduce the whole team's outcome at a net personal cost. Second, contributing to the public good more than others is costlier in the WLM, as no additional public good is created. But, low contributors receive no benefit from high contributions, as the team's output is only determined by the minimum contribution. In other words, low contributors cannot free ride on high contributions. Croson et al. (2005) and Fatas et al. (2006) characterize these two games.

Introducing a non-trivial equilibrium selection problem, the WLM is a much richer strategic environment. Interestingly, individuals still get the same earnings in both environments if they all contribute the same proportion of their endowment. Each symmetric contribution profile yields the same earnings in both games. The difference is that symmetric contribution profiles are equilibria of the stage game only in the WLM. Being the strategy space of both games quasi-continuous, the equilibrium selection problem is far from trivial in the WLM. Full contribution Pareto dominates all other actions, while no contribution satisfies the maximin criterion and risk dominates the other equilibria of the game, minimizing losses associated to strategic uncertainty^3^.

From the group's fitness perspective, contributing to the public good is now riskier because contributions above the minimum do not increase the collective account benefits. Strong contributors may totally waste their contributions if another player in their group does not contribute to the public good. Punishment coming from strong cooperators may still be considered as social punishment as long as it promotes more cooperation and contributes to the collective or social welfare (but not because it sanctions free riding). Punishment implemented by low contributors when punished may still be defined as antisocial, as it might be highly detrimental for group cooperation.

The interaction between different types of punishment and the effect of sanctions on group fitness through incentives is far from trivial when no information about the identity of the punisher is provided, as in this study. Incentives are the same if an intermediate cooperator is punished by a complete free rider or by a full cooperator. However, in this paper we explicitly follow an agnostic view and consider that even if participants never know who is punishing them, different types of punishment may (or may not) have first order effect on group fitness through different levels of effectiveness. Following Herrmann et al. (2008), our paper does not try to prove causation; it simply investigates the existence of different logics in both games supported by the very different levels of social and antisocial punishment.

**Figure 1:**
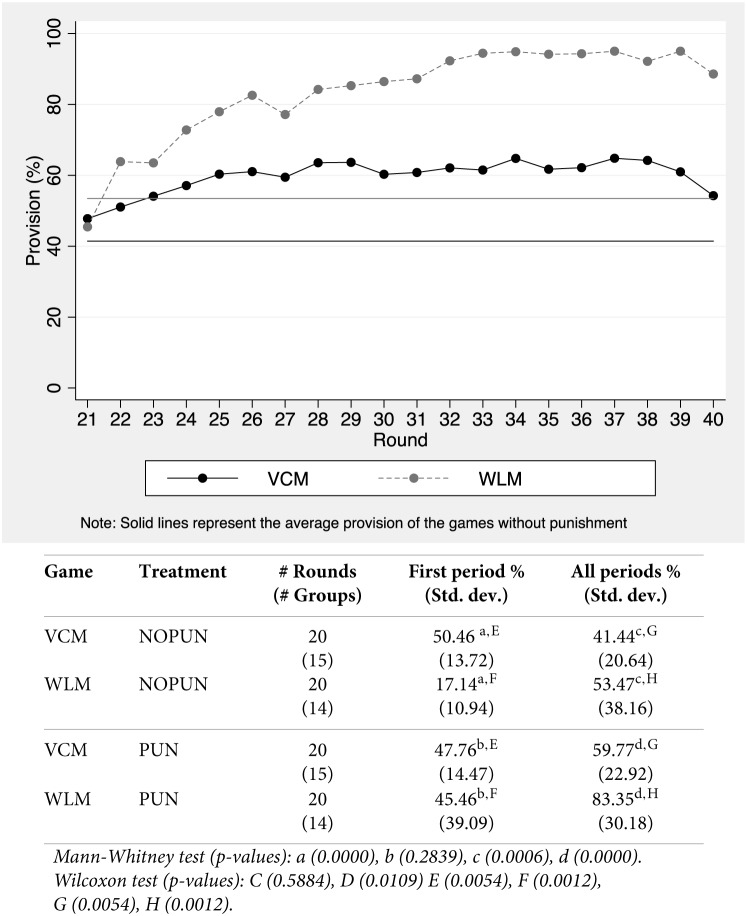
**Provision of the public good**.

**Figure 2:**
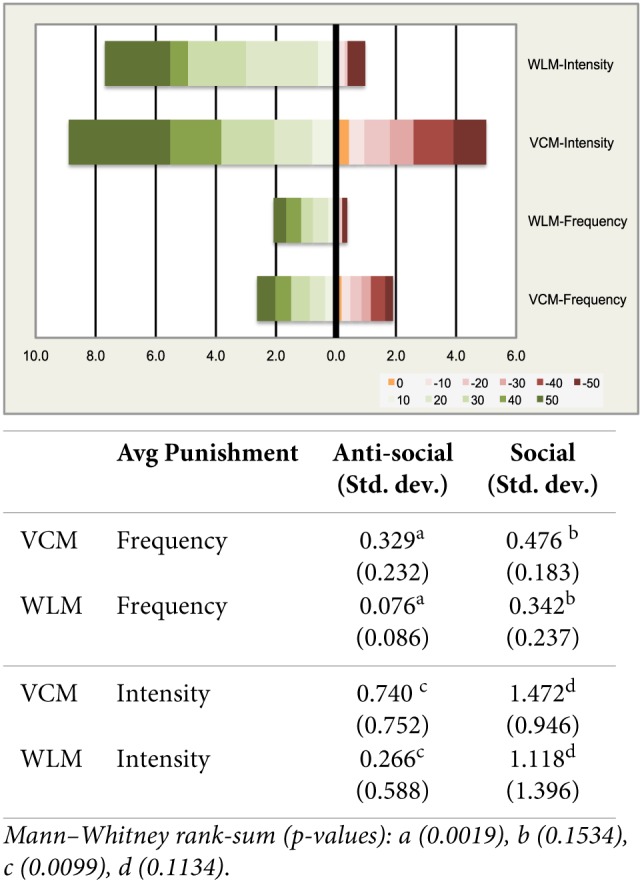
**Social and Anti-social punishment**.

In this paper we explore the preeminence of social and antisocial punishment in both public goods games in a particular location: Spain. Gächter and Herrmann (2009) and Gächter et al. (2010) follow both Inglehart and Baker (2000) and the four cultural dimensions of Hofstede (2001) to classify countries in different cultural areas. On the basis of the results obtained in Greece and Turkey, Southern Europe is defined as a region with a very moderate level of contributions to the public good, when sanctions are available (see Figure 1 in p. 2655) and a quite high level of antisocial punishment (see Figure 2 in p. 2656). Casari and Luini (2009) shows that in standard VCM games, Italian subjects exhibit considerable antisocial punishment, Bortolotti et al. (2014) report evidence of social and antisocial punishment in a sample of college students and a representative sample of the Italian population playing a VCM game. Fatas et al. (2010) and Alonso and Gächter (2014) show that linear public goods games in Spain follow a very similar pattern. In computerized anonymous experiments run in Valencia and Granada, respectively, they find that contributions to the public goods never reach very high levels with punishment, and Alonso and Gächter (2014) find that antisocial punishment is as strong as in Greece or Turkey as it is in Granada.

We run our experimental sessions in Valencia, Spain. We are particularly interested in replicating, first, the prevalence of antisocial punishment in linear public goods games (the VCM) and willing to learn whether punishment follows a similarly destructive dynamic when the production technology is non-linear.

## Experimental design and procedures

### The experiment

Our experiment consists of two games and two treatments. As described above, the two games are the *voluntary contribution mechanism* (VCM) and the *weakest link mechanism* (WLM)^4^. Subjects interact over a computerized network of computers in two blocks of 20 rounds each. So, for each treatment and game, subjects played a finitely repeated public goods game for 20 periods. We opt for this relatively long sequence of repeated interactions because we are interested in testing the long term effects of sanctions, see Alonso and Gächter (2014) for an interesting review of this literature. We introduced a surprise restart^5^ in the games at the end of the first block, when costly punishment is introduced. Participants from the large local database were recruited electronically and randomly assigned to treatments within the experiment, sessions within each treatment and cubicles within each session. Individuals were undergraduate students with different social sciences backgrounds and had no experience in these games, and participated in only one session.

In our study, the composition of each group remained unchanged throughout the experiment (using a *partner* protocol). All participants were aware that each block would last exactly 20 periods. However, they were not aware that a second treatment was to follow^6^. Table 1 summarizes the number of participants, the independent observations, the production function and the Nash equilibria predicted for each experimental manipulation.

**Table 1:** **Experimental design**.

	**VCM**	**WLM**
Block 1	No punishment	No punishment
Block 2	Punishment	Punishment
Subjects	60	56
Groups	15	14
Production function	Linear	Minimum

#### VCM

Our first treatment is the standard *voluntary contribution mechanism* as presented first by Isaac et al. (1984). VCM games are the most common in public goods settings (see Ledyard, 1995; Keser, 2002, or Chaudury, 2011, for reviews). The amount of public good provided depends on the sum of individual contributions to the public good. Each round, participants received a fixed endowment of 50 Experimental Currency Units (ECUs) and had to decide how many ECUs to keep in their private account and how many to allocate to a collective account. All the participants made their decision simultaneously and anonymously. The individual payoff function is then:

(1) $$\pi_{i}=\left(e-c_{i}\right)+b\;\cdot \;\sum_{i\;=\;1}^{n}c_{i}=\left(e-c_{i}\right)+b\;\cdot \;n\;\cdot \;avg(c_{i})$$

where *e* is the endowment in ECUs, *c_i_* the team account investment, and *b* the marginal per capita return (MPCR) from the project (0.5 in our design). For convenience, the payoff function was presented to subjects as a function of the relevant statistic (the average).

As usual in these games, allocation decisions have monetary consequences. Participants' monetary compensation depends on the benefits individuals get from their private account (the ECUs they keep for themselves), as indicated by the first term of the equation [1], and from an equal share of the team account's profit (second term of the equation). Equation [1] also implies that full free riding (*c*_*i*_ = 0, π_*i*_ = 50) is a dominant strategy in the stage game^7^. However, the aggregate payoff is maximized if each group member fully contributes to the public good (*c_*i*_ = 50, π_*i*_ = 100*). Note that with groups of four players, these parameters generate round numbers and make it relatively easier for participants to understand the consequences of their actions.

#### WLM

We use in our second treatment a variant of the *weakest link mechanism* as studied in Weber et al. (2004). In this particular game, the amount of public good provided depends on the minimum allocation to the group account. Participants receive a fixed endowment of 50 (ECUs) at the beginning of each round, and have to simultaneously and anonymously decide how many ECUs to allocate to the group account. The individual payoff function is then:

(2) $$\pi_{i}=\left(e-c_{i}\right)+b\;\cdot \;n\;\cdot \;min(c_{i})$$

Note that the laboratory protocol used in our WLM is essentially identical to the one described above for the VCM. Individuals are randomly assigned to groups of size four, and each individual received the endowment to allocate between the two accounts, also neutrally framed as an allocation problem. Participants earn the sum of their allocations to the private account and twice the minimum allocation to the public account. Given that participants are able to choose any contribution up to one decimal, the stage game has 501 Nash equilibria in pure strategies. Each symmetric contribution profile is equilibrium of the game. In the Pareto efficient equilibrium everybody allocates her endowment to the public account (*c_i_* = *50* and π_*i*_ = *100*), and the equilibrium in which none contributes to the public good satisfies the safe *maximin* criterion (*c_i_* = *0* and π_*i*_ = *50*).

The WLM has been used to capture features of joint production with strong complementarities (Weber et al., 2004), or coordination failure in organizations (Brandts and Cooper, 2006). In their seminal paper, Van Huyck et al. (1991) analyzed minimum effort coordination games with seven symmetric Pareto-ranked equilibria. Participants repeatedly played different versions of the game. The selection of the payoff-dominant equilibrium was extremely unlikely in almost every single experimental condition. A quite active branch of the literature searched for different ways to improve efficiency in these games (that is, reaching better coordination on Pareto superior equilibria). Some successful possibilities are to reduce strategic uncertainty (Van Huyck et al., 1991), adding competition within or between groups (Riechmann and Weimann, 2008; Croson et al., 2015), tacit communication (Cachon and Camerer, 1996; Broseta et al., 2003) or sequential stages (Weber et al., 2004).

#### Punishment

In the second block of 20 rounds, we introduced a second stage of punishment in both the VCM and the WLM. As in Fehr and Gächter (2000), we use both a within- and a between-subjects design. All participants went through the same sequence of games: first without punishment and then with punishment (*within-subjects*), and they only participated in one experiment (*between-subjects*).

In the punishment stage subjects had the opportunity to simultaneously assign punishment points to the other group members, after being informed of their individual contributions. Each punishment point reduces both the earnings of the sender and the receiver (in 2 and 6 ECUs, respectively, using the standard fixed rate technology, as in Herrmann et al. (2008) and Nikiforiakis and Norrmann, 2008). That is, punishment was costly not only for those participants punished, but also for any player *sending* punishment points. Relative to Herrmann et al. (2008), we scale up the cost of punishment for both players because our participants receive 50 ECUs as endowment, rather than 20. By doing so, we keep the ratio between the cost of sending and receiving punishment points (1:3). Note that losses from punishment could exceed the preliminary profits, and losses were compensated across rounds.

In all treatments and blocks, participants were told at the end of each round the individual contributions profile^8^, their own earnings both in total and coming from both the private and public accounts, and their preliminary and final profits after the punishment stage, if available. Participants in our study were University of Valencia social science undergraduate students and had no previous experience in similar experiments. Participants were electronically recruited and were privately paid at the end of the experiment. Experiments were run at the experimental laboratory of the University of Valencia, using z-Tree (Fischbacher, 2007) in 2010 and 2011, and followed the standard methodology in experimental economics (including full anonymity and privacy). Experiments took less than 90 min, and the average earnings were 18€.

## Experimental results

We focus in the analysis of punishment, starting with its effectiveness. Figure 1 presents the round provision of the public good (the size of the team output), for both games in the second block of 20 rounds with punishment, normalized as a percentage of the maximum (see the lines with markers). As a reference point, we also add the average provision in the 20 rounds without punishment (straight lines with no markers). Below Figure 1 we present some descriptive statistics of provision as percentages of the maximum provision level, together with the results of simple and conservative non-parametric tests^9^.

In line with Croson et al. (2005, 2015), differences across the two games without punishment are secondary. Provision in the first period of the WLM is clearly lower than in the VCM (50% vs. 17%, see Mann–Whitney tests^10^ in Table 2), because coordination is rare without interaction^11^. Differences become only marginally significant for the 20 rounds, and are of a much smaller magnitude. In line with Alonso and Gächter (2014), provision with full individual information is moderate in the VCM (41.44% of the maximum) and slightly higher in the WLM (53.47%).

When punishment is introduced in the second block, provision in round 21 is almost identical in both games (and not significantly different from each other, 48% vs. 45%). The introduction of punishment generates significant cooperation gains in both games, as provision with punishment is substantially larger than in the first block: up to 59.77% and 83.35% of the maximum in the VCM and the WLM (significant at the 1% level running a Wilcoxon sign-rank test).

Figure 1 clearly suggests punishment has very different effects on the provision of the public good in the VCM and the WLM. While punishment takes provision close to its maximum in the WLM (an striking average of 83%), the impact in the VCM is flatter and more moderate. The asymmetric effect of punishment is confirmed by non-parametric tests: provision is higher in the WLM than in the VCM in the second block as a whole (Mann–Withney test, *p*-value < 0.000). Provision in the last round of the block is significantly higher than in the first round in the WLM (Wilcoxon signed rank test, *p*-value< 0.0025) but not in the VCM (*p*-value < 0.6089).

Note that the one interpretation of this result could be that the WLM is an *easier* social dilemma than the VCM. If this interpretation were true, a *little help* from informal punishment would suffice in one game (the WLM) to increase efficiency, but not in the other. We discard this interpretation because if true, we would expect our participants to do significantly better in the WLM than in the VCM when punishment is not available. In line with Croson et al. (2005) and Croson et al. (2015), they do not.

As Figure A_1 in Appendix II (Supplementary Material) suggests, provision stays stable in the second 10 rounds of the WLM without punishment, and no significant differences are found between both games without sanctions. Moreover, only when punishment is available efficiency significantly increases in the WLM, while it does not in the VCM. As the vast and more general experimental literature on coordination games seems to suggest, we also believe there is not much previous support for the idea that coordination games with multiple equilibria are simpler than linear social dilemmas. If any, and starting from Van Huyck et al. (1991), coordination games seem to be extremely context dependent.

We specifically try to address this context dependent issue by showing how in one very particular context (identical for both games), sanctions are significantly more effective in the WLM than in the VCM, even when the number of equilibria is much higher than in the standard minimum game. More than that, we articulate a rationale based on the negligible impact of antisocial punishment in one game (the WLM) and not in the other (the VCM)^12^.

We analyze now the effectiveness of sanctions paying distinctive attention to the behavioral patterns of social and antisocial punishment in the two games. Following Herrmann et al. (2008), we define social (antisocial) punishment as the one imposed on subjects contributing less (more) than the sender. Figure 2 below shows social and antisocial punishment as a function of the distance between the contribution of the punisher and the contribution to the team account of the receiver (punished). The distance can be as high as +50 (if the participant sending punishment points contributed 50 ECUs and the participant punished 0) and as low as −50 (if the participant sending punishment points contributed 50 ECUs less). Following Herrmann et al. (2008), we group observations in intervals of 10 units.

We measure both the frequency and the intensity of social and antisocial punishment in the VCM and the WLM. We define frequency as the proportion of positive punishment events (from 0 to 1, in each interval), and we define intensity as the average number of punishment point sent (rom 0 to 10, in each interval. By inspection, Figure 2 shows that in line with the classification of different cultures made by Gächter and Herrmann (2009) antisocial punishment is intense in the VCM in Spain, as part of the Southern European culture. Even when social punishment is also stronger in frequency and intensity in the VCM, the magnitude of antisocial punishment is consistent with their explanation about the weak effect of sanctions in some cultures. Provision of the public good does not exhibit a positive trend (no differences between round 21 and round 40), and it fails to reach 60% of full provision because antisocial punishment harms the positive effect of sanctions.

Interestingly, the WLM looks quite different in our study. While no significant differences in the frequency and intensity of social punishment are observed when comparing the VCM and the WLM (47.6% vs. 34.2% and 1.47 vs. 1.12), antisocial punishment clearly diverges in the two games (32.9% vs. 7.6% and 0.74 vs. 0.27). In order to give these comparisons a statistical support, we compute the average frequency and intensity of social and antisocial punishment per group (pooling the different types of social punishment and the different types of antisocial punishment). We find that antisocial punishment is less frequent in the WLM than in the VCM (Mann–Whitney rank-sum *p*-value < 0.0019) and is significantly less intense (*p*-value < 0.0099) (15 observations in the VCM; 14 observations in the WLM). No significant differences are obtained when running the same comparison for social punishment.

In summary, we observe that punishment is not only less frequent and less intense in the WLM in our experiment, but also more asymmetric. When actions are complementary, as in the WLM, punishment is highly effective generating full provision of the public good even in Southern Europe. Consistent with Herrmann et al. (2008) interpretation, punishment is highly effective when antisocial punishment is rare, and not intense^13^. We now try to understand how this asymmetric punishment pattern translates into contributions to the public good and efficiency.

**Figure 3:**
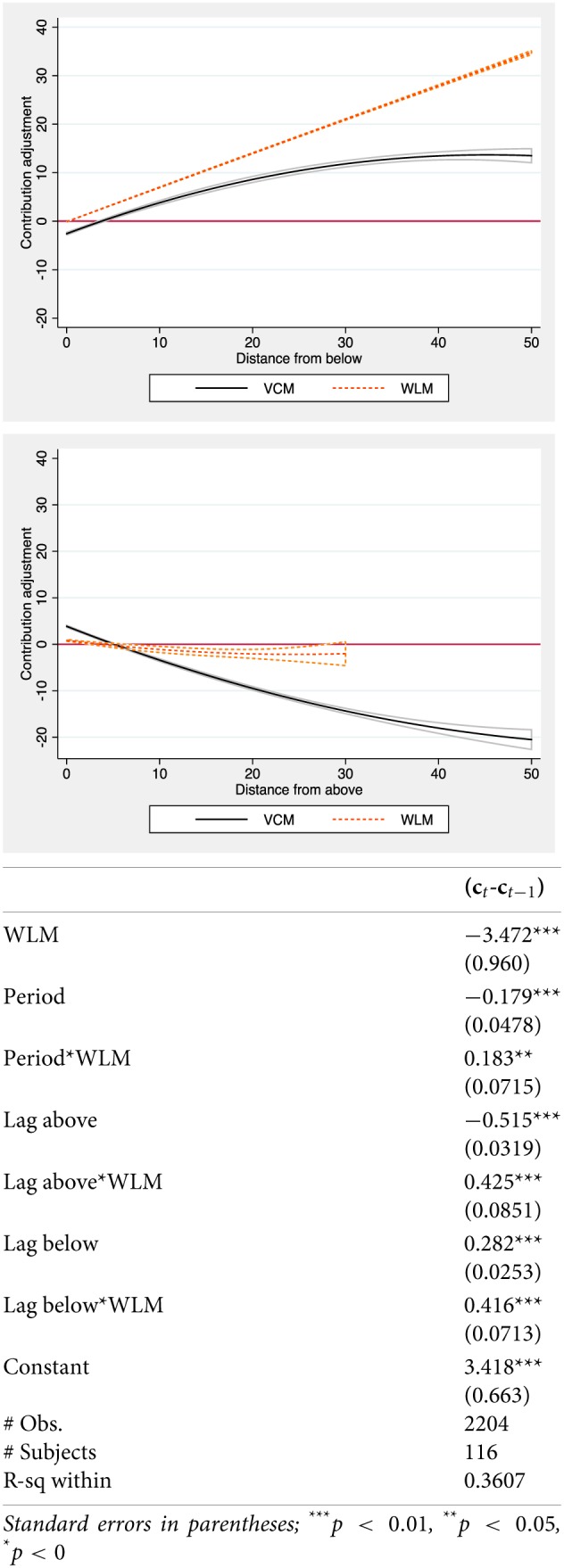
**Contribution adjustment**.

We now know that punishment decisions significantly differ across games. We need to understand what is the differential effect of social and antisocial punishment in the WLM. In order to understand the behavioral implications of sanctions on public good provision, we run a Tobit regression with the adjustment of individual contributions over time (*c_*t*_-*c*_*t* − 1_*) as dependent variable (presented in Figure 3, below). We introduce random effects at the individual level, cluster standard errors at the group level, and focus in the second block of 20 rounds, with punishment. The independent variables in the analyses try to explain contribution changes as a function of the relative position of subject i's decisions, and include one treatment dummy (*WLM*: 1 for the WLM and 0 for the VCM), *Period*, and two variables indicating the lagged difference between subject i's contribution and the average contribution of the other subjects in the same group, *Lag above*, and *Lag below*, when the difference is positive (negative), zero otherwise.

These two variables allow us to understand how sanctions shape contribution adjustments when participants are contributing more (less) than the rest of the group; that is, in the position of becoming social (antisocial) punishers. Additionally, we introduce interaction terms (Period^*^WLM, Lag above^*^WLM and Lag below^*^WLM) to capture differences across games^14^. In this sense, we can isolate the effect of independent variables in the WLM.

Note that, following Herrmann et al. (2008), Figure 2 uses the relative position of individual participants to classify punishers (as social or antisocial). Following most of the literature on conditional cooperation (see Croson et al., 2005) we classify contribution adjustments in Figure 3 using the mean decision of the other participants in the same group. Punishment points may be send individually, after observing every individual contribution, and each participant may or may not punish each other group member independently. Participants may only adjust their contributions once, as a reaction to the contribution of the others. So, it becomes impossible to use individual decisions as a reference point in Figure 3, and it is more meaningful to consider any contribution change using the mean contribution of the rest of the team.

For the sake of clarity, Figure 3 plots the predicted contribution adjustments of the model below when contributing more (less) than the rest of the group, across the two games considered, allowing for any non-linearity and including the standard confidence intervals^15^. The horizontal axes represents the absolute distance (from below or above) and the vertical axes refer to the contribution adjustment, as explained. The table below Figure 3 presents the coefficients of the estimated model.

Differences between both games are well-defined. Participants modify their contributions similarly when contributing less than the rest of the group: they adjust up their contributions, and their reaction is more intense the larger the distance between their contribution in (t-1) and the contribution of the rest of the group. Interestingly, as the significant coefficient of the interaction term suggests (Lag below^*^WLM), the adjustment is more intense in the WLM.

Differences across games are more intense when participants contribute more than the others. Consistently with standard conditional cooperation patterns, participants exhibit the usual negative adjustment when contributing more than the rest of the team in the VCM. From above, participants in the VCM significantly adjust down their contribution, and the intensity of the adjustment increases with the distance between their lagged contribution and the lagged contribution of the rest of the group. In the WLM, this adjustment pattern simply disappears: participants contributing more than the others do not adjust down their contributions. As the flat figure and the positive interaction term (Lag above^*^WLM, cancelling out the negative *Lag above* coefficient) strongly suggest, the different use of antisocial punishment is consistent with an interesting shift of conditional cooperation patters. Figure 2 suggested that when contributing relatively less, participants in the WLM did not exhibit antisocial punishment. Figure 3 explains that, when contributing relatively more, participants in the WLM pay back by not adjusting down their contributions^16^.

**Figure 4:**
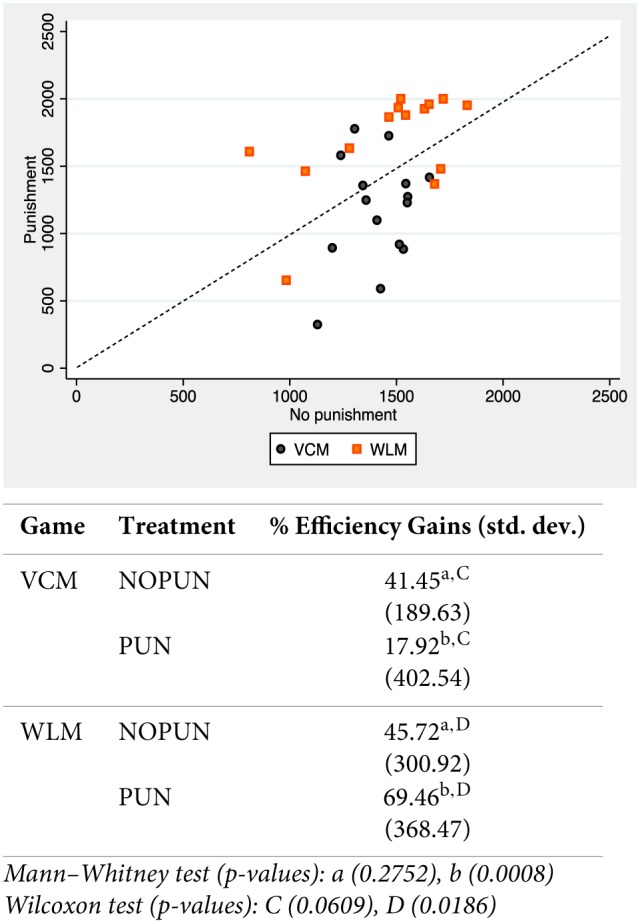
**Efficiency**.

We conclude this section by studying the consequences on efficiency, using earnings as a proxy. This discussion is always present when considering the net fitness effect of sanctions in public goods experiments. As punishment is costly for both punisher and punished participants, any conclusion on fitness gains has to consider the welfare effects of sanctions, net of any punishment costs. In Figure 4 we plot absolute earnings with and without punishment at the group level (earnings without punishment in the horizontal axis, and earnings with punishment in the vertical axis). We add a 45 degrees line to make the comparison easier. We also include the results of non-parametric tests at the bottom of the figure^17^.

Not surprisingly, Figure 4 shows that the asymmetric punishment patterns and the different cooperation adjustment translate into very different efficiency gains. While most of the groups in the WLM do better (above the 45° line) with punishment (11 out of 14), the opposite result is observed in the VCM (11 out of 15 groups do worse). These results are supported by the non-parametric tests shown below, using a linear transformation of earnings into efficiency gains (as discussed above, earnings above the no-contribution level): no differences are observed without punishment (41.45 vs. 45.72, *p*-value < 0.2752), but differences are extremely large and significant with punishment (17.92 vs. 69.46, *p*-value < 0.0008). Interestingly, earnings significantly increase with punishment in the WLM, *p*-value < 0.0186 (marginally decrease in the VCM, *p*-value < 0.0609).

## Discussion

Costly punishment has substantial effects in human cooperation. In this paper we present the results of a sequence of experiments run in Southern Europe using two different public goods games. When the production technology is linear, as in the VCM, punishment has only a small positive effect on contributions, hindered by a substantial amount of antisocial punishment. When the production technology is non-linear, as in the WLM, public good provision is significantly increased by punishment, and contributions go up to 95% of the endowment at the end of the experiment. In this sense, punishment in our experiment has a stronger positive effect in the WLM, as both the trend and the absolute levels of public good provision are significantly above the ones observed in the VCM.

Our data provides a rationale for this difference, as participants in our study use punishment in very different ways, and when used, it generates very different behavioral reactions. While social punishment is indistinguishable in the two games, antisocial punishment is rare, and remarkably less intense in the WLM. The moderate use of antisocial punishment generates a positive effect on contribution adjustments, and the conditional cooperation of top contributors is reinforced by the lack of antisocial punishment: contributors do not adjust down their contribution to the contribution of others, boosting provision, and earnings.

Our experiments were run in a location where the effectiveness of punishment tends to be moderate. We replicate this findings when the production technology of the public good is linear, but completely fail to replicate this result when the technology follows a weak-ling dynamic and contributions to the public good are complementary. Given that our study was run only in one location, we cannot infer much about the effectiveness of sanctions in other locations when using the same weak link technology. However, under the realistic assumption that the production technology of public goods does not have to be unique, our study significantly contributes to the debate on the co-evolution of cooperation norms and punishment.

The very moderate use of antisocial punishment in the WLM shows that the connection between cooperation norms and sanctions may critically depend on the team production technology. Our results suggest that the portability of the vicious circle linking antisocial punishment with weak cooperation norms and poor group performance could be limited to particular types of public goods, and non-necessarily replicable in other forms of group interactions.

## Author contributions

Enrique Fatas contributed to the original study design, run the experimental sessions, interpreted the data, wrote the final versions of the manuscript. Guillermo Mateu assisted running the sessions, assisted in the interpretation of the data, and wrote the first draft under the supervision of Enrique Fatas.

### Conflict of interest statement

The authors declare that the research was conducted in the absence of any commercial or financial relationships that could be construed as a potential conflict of interest.

## Footnotes

^1^We follow closely the spirit of Herrmann et al. (2008), because we are specifically interested to replicate their study in a location where sanctions did not substantially increase cooperation when information about individual punishment decisions was not available, as discussed in the next page.

^2^As the results section makes clear, using any of the two definitions of cooperators in anti-social punishment (those above the average contribution in the group, or above the contribution of the individual punishing) do not affect our results, as Table A_7 in Appendix II (Supplementary Material) strongly suggests. In line with Herrmann et al. (2008), we will use the latter in the analysis of social and antisocial punishment.

^3^In Appendix II (Supplementary Material)we present an analysis of equilibrium selection in the WLM and symmetric contribution profiles in the VCM. As it will be noted in Section Experimental Results, a detailed analysis of equilibrium selection is not the main goal of the paper, even when punishment intensity is very different when participants are in or off-equilibrium.

^4^In both cases, contributions are voluntary. We use the term VCM for convenience, in line with previous studies, as noted in Section Literature Review.

^5^The surprise- restart technique has been investigated in repeated public goods experiments (see, e.g., Andreoni, 1988; Croson, 1996, 2000)

^6^As in Herrmann et al. (2008), we are not particularly interested in the analysis of order effect. As Fatas et al. (2010) show, and in line with the original findings of Fehr and Gächter (2000), the results are very similar if the sequence of games is reversed.

^7^As Table A_2a and Figure A_2b in Appendix II (Supplementary Material) makes clear, the inefficient equilibrium is almost never selected without punishment (80% of the groups never reached it across the first 20 rounds of the experiment, without punishment, and it is only selected 2.3% of the times by all groups across these 20 rounds).

^8^Decisions were showed identifying the number of the player within the group. Thus, subjects can identify the contribution of each member of the group, this allow them to punish each other identifying contributions. As it will be noted below, this amount of social identification generates a moderately high level of provision even without punishment, in line with Alonso and Gächter (2014). Retaliation here can only be blind since each participant is never informed of the punishment that each individual assigned to him.

^9^Note that we present the provision of the public good rather than the average decision. While allocation and provision coincide in the VCM, in the WLM the provision of the public good is determined by the minimum allocation to the group account. Following Herrmann et al. (2008), we are specifically interested in the effectiveness of sanctions in increasing group fitness, or welfare. Even when the results are not very different when we plot actions, and we specifically study the adjustment of decisions later in this section, we believe Figure 1 captures well the main message of this paper. The curious reader may have a look at the evolution of provision without punishment in Appendix II (Supplementary Material), Figure A_1.

^10^We use a Mann-Whitney rank sum test (at the group level) to analyze the differences between games, and Wilcoxon signed rank tests to analyze differences within games across treatments (punishment vs. non punishment). We only take one observation per group, treatment and game.

^11^Note that two vectors of decisions with the same mean and a positive variance logically generate less provision in the WLM.

^12^Note that effort choices are related to group fitness and efficiency only in the VCM. An analysis of effort has to be completed by incorporating efficiency gains, defined as earnings above the no contribution profile, when none contributes to the public good, and no group externality is generated. Efficiency gains balance the benefits of provision with the costs of miscoordination, hidden if only effort is considered. To prove this point, consider the following example, constructed in an scenario without sanctions. In both games, 3 individuals contribute 40 and the other one contributes zero. Identical effort profiles generate the same average effort (30). However, provision and efficiency gains (as defined above) dramatically differ. While provision is 30 out of a maximum of 50 in the VCM, provision of the public good is 0 in the WLM. Interestingly, participants are obtaining substantial efficiency gains in the VCM (average earnings are 80, 30 above the no-contribution profile) and identical efficiency losses in the WLM (average earnings are 20, 30 below the minimum symmetric payoff of 50), because of the large miscoordination cost. Figure A_5 in Appendix II (Supplementary Material)shows effort and efficiency gains in both games. Interestingly, punishment generates large efficiency gains in the last ten rounds with punishment only in the WLM, in which miscoordination is negligible and punishment is rarely used.

^13^Tables A_2a and A_2b analyze effort across games (VCM and WLM) and conditions (No punishment and Punishment) dividing the data into two categories: symmetric profiles (when all group members make the same decision in a given round) and asymmetric profiles. As any symmetric contribution profile is an equilibrium of the stage game in the WLM, the division could be informative in the analysis of a different punishment effects in the two games. In line with Figure 2 above, effort in symmetric profiles are significantly higher than those observed in asymmetric profiles, with and without punishment, only in the WLM. In the VCM, symmetric effort profiles are lower than asymmetric ones without punishment, and higher when sanctions are available. Table A_3a and A_3b in Appendix II (Supplementary Material)show a similar pattern in efficiency gains. The equilibrium selection problem does not invalidate Figure 3, as sanctions do not alter the number of equilibria in the WLM, and symmetric profiles always generate significantly more effort and efficiency gains.

^14^These variables capture the interaction between the independent variables and the treatments dummy.

^15^Figure 3 is generated using the *qfitci* Stata command on the predicted contribution adjustment.

^16^Table A_7 in Appendix II (Supplementary Material) includes an analysis of how punishment intensity follows two different reference thresholds as proxies of the social norm: model 1 (model 2) explains punishment intensity as a function of the distance between the contribution of the punisher and the group average (the contribution of the individual punished). Left censored Tobit models strongly suggest that sanctions are significantly less intense in the WLM and that both reference points yield very similar results. Tables A_6a, and Figure A_6b show that punishment is very rare when all decisions are identical in the group, both in the VCM and the WLM, reinforcing the idea that punishment is mostly driven by differences in contributions.

^17^For the sake of clarity, the table refers to efficiency gains, normalized from 0% (if no contribution is made to the public good) to 100% (full contribution and no punishment)

## Supplementary material

The Supplementary Material (Appendix I contents the experimental set of instructions and Appendix II adds extra tables and figures) for this article can be found online at: http://journal.frontiersin.org/article/10.3389/fnbeh.2015.00107/abstract
