# Supplementary material for: Antisocial punishment in two social dilemmas
Source: Front Behav Neurosci. 2015 Apr 29;9:107. doi: 10.3389/fnbeh.2015.00107 (PMC4413789; doi:10.3389/fnbeh.2015.00107)
Supplement: Supplementary file 1 [file DataSheet1.DOCX]

**Appendix I**

*Instructions (translated from Spanish, without punishment, for both games, WLM between brackets and in italics)*

1. The experiment has 20 rounds. Each round you make decisions in a group of 4 participants. At the beginning of the experiment you will be randomly matched with three other participants in a group, and the composition of the group will not change throughout the experiment. You will never know the identity of the other participants in your group.
2. Each member of the group is randomly assigned with one number in the group (1, 2, 3, 4); this number will remain constant during the experiment. Each group has the same Participant 1, Participant 2, Participant 3 and Participant 4. You will be one of these participants.
3. At the beginning of each round, each participant will receive an initial endowment of 50 ECUs. Your unique decision consists on choosing the quantity of ECUs you want to allocate to a Collective Account (CA). The rest of ECUs will be automatically allocated to an Individual Account (IA).
4. The profit from the Collective Account is calculated computing the sum of all [*WLM: the minimum*] allocations to the Collective Account in your group (that is, the sum of all [*WLM: the minimum*] allocations of participants 1, 2, 3 and 4). [*Only in the WLM: The minimum is multiplied by four, as if every group member allocated the minimum to the Collective Account*]. The result is doubled and equally shared by the four group members. Note that this is equivalent to say that the profit from the Collective Account is twice the average [*WLM: the minimum*] allocation to the Collective Account in your group.
5. The profit from the Individual Account is the allocation to this account and does not depend on the decisions of the other participants.
6. To summarize, your profit each round will be determine by:

Total profit = Profit (Collective Account) + Profit (Individual Account)

(2x average [*WLM: minimum*] allocation) (50-allocation to CA)

1. Each round you will receive information about the allocations of the other participants in your group to the Collective Account. At the end of each round, you will also receive information about your past decisions and profits.

**Appendix II**

**Figure A_1. Provision of the public good without punishment**

Figure A_1 shows that the provision of the public good in the two games is not substantially different on average (a Mann Whitney test shows marginally significant differences across games; p<0.0668), even when the different dynamics are apparent. Interestingly, the VCM without punishment shows the moderate decline observed in some locations with weak norms of civic cooperation (see Hermann et al, 2008). In contrast, the WLM shows a positive trend in the first ten blocks (when individuals learn to mitigate miscoordination), and a remarkably flat plateau in the second ten rounds, when provision gets stacked slightly above 60% of the maximum level. A standard analysis of trends (using panel data estimation with random effects, and one observation per group) shows that period is significantly positive in the first ten rounds and not significantly different from zero in the second.

**Table A_2a: Symmetric profiles frequency (%) – Descriptive statistics and non-parametric tests**

|  | No punishment | Punishment |
| --- | --- | --- |
| VCM | 2.67 **^a^ ^c^** | 11.33 **^b^ ^c^** |
| WLM | 36.43 **^a^ ^d^** | 70.71 **^b^ ^d^** |

a, b, c, d all Fisher exact test p-values<0.0000

**Figure A_2b: Symmetric profiles – Distribution by group across rounds, by game and condition (punishment and non-punishment)**

Table A_2a shows the distribution of symmetric decisions profiles across games (VCM and WLM) and conditions (No punishment and Punishment). By a symmetric contribution profile, we refer to a situation in which all group members allocate the same amount to the team account, denoted as the chosen effort or the contribution to the public good in the text. For the sake of comparability, we analyze all symmetric profiles, even when in the VCM only the fully inefficient zero profile is an equilibrium of the stage game. Table A_2a shows that symmetric profiles are significantly less frequent in the VCM than in the WLM and that punishment significantly increases these proportions in both games (as the outcome of the different exact Fisher tests strongly supports).

Figure A_2b shows these differences by presenting the different group types: in the horizontal axis the group frequency may go up form 1 (participants in one group always choose a symmetric effort profile in the 20 rounds of the block) to 0 (participants in one group never choose a symmetric profile). Figure A_2b clearly shows that while 80% of the group sin the VCM never contribute a symmetric profile without punishment, and not a single group submits a symmetric decision profile more than 30% of the times, punishment significantly increases the likelihood of symmetric play: 40% of the groups submit a symmetric profile at least once, and one group doe sit more than 70% of the rounds. Symmetric profiles, all of them equilibria of the stage game, in the WLM are much more frequent, and the distribution also shifts to the right when sanctions are introduced in the second 20 rounds.

**Table A_3a: Effort – Descriptive statistics and non-parametric tests**

|  | Symmetric profiles | | Asymmetric profiles | |
| --- | --- | --- | --- | --- |
|  | NP | PUN | NP | PUN |
| VCM | 6.25 | 37.94 **^e^** | 21.12 ^f^ | 28.86 **^g^** |
|  | (16.80) | (19.25) | (15.72) | (15.98) |
| WLM | 41.68 | 49.77 **^e^** | 24.27 ^f^ | 30.36 **^g^** |
|  | (15.78) | (2.27) | (16.93) | (15.36) |

Two-sample Wilcoxon rank-sum (Mann-Whitney) tests

e. pvalue<0.0197 (n=6+13); f. pvalue<0.2217 (n=15+14); g. pvalue<0.0188 (n=15+11)

**Table A_3b: Effort – Data panel regressions**

|  | (1) | (2) | (3) | (4) |
| --- | --- | --- | --- | --- |
| Effort | VCM - NP | VCM - Pun | WLM - NP | WLM - Pun |
| Period | -0.424*** | 0.219 | 0.781** | 0.335** |
|  | (0.118) | (0.227) | (0.316) | (0.161) |
| Symmetric profile | -7.584 | 0.428 | 6.673*** | 10.63*** |
|  | (6.330) | (7.495) | (2.503) | (1.841) |
| Constant | 25.38*** | 27.54*** | 19.98*** | 33.05*** |
|  | (1.907) | (2.471) | (3.659) | (3.390) |
| Observations | 1,200 | 1,200 | 1,120 | 1,120 |
| Number of subjects | 60 | 60 | 56 | 56 |

Robust standard errors in parentheses

*** p<0.01, ** p<0.05, * p<0.1

Table A_3a presents some descriptive statistics of effort choices across games and conditions (including means and standard deviations between brackets), dividing the whole sample in symmetric and asymmetric contribution profiles. The non-parametric tests show that (i) decisions are different when aligned (as in the symmetric effort profiles), and (ii) the effect of punishment on effort choices is heavily mediated by the game characteristics.

Effort is significantly higher when decision choices are the same in the WLM. Both with and without punishment, effort goes up by roughly 50%, and in a significant way (both pairwise comparisons are significant at least at the 5% level, according to the Wilcoxon signed rank test, using one observation per group, whenever the group is at least once in a symmetric position). However, the effect is very different in the VCM. Even when symmetric profiles are so rare in the VCM that it is impossible to run a solid non parametric statistical test, symmetric effort profiles are lower without punishment (most likely driven by the attraction of the unique inefficient equilibrium) and higher with punishment.

Table A_3b presents the outcome of simple panel date regressions with random effects at the individual level and standard errors clustered at the group level, and find a large, negative, but insignificant coefficient for the dummy symmetric profile in the VCM without punishment, and large and positive differences in the WLM, both with and without punishment.

**Table A_4a: Efficiency gains – Descriptive statistics and non-parametric tests**

|  | Symmetric profiles | | Asymmetric profiles | |
| --- | --- | --- | --- | --- |
|  | NP | PUN | NP | PUN |
| VCM | 6.25 | 26.94 **^h^** | 21.12 **^i^** | 6.66 ^j^ |
|  | (16.80) | (34.43) | (15.72) | (26.90) |
| WLM | 41.68 | 47.82 **^h^** | 12.08 **^i^** | 3.12 ^j^ |
|  | (15.78) | (10.62) | (19.86) | (31.53) |

Two-sample Wilcoxon rank-sum (Mann-Whitney) tests:

h. pvalue<0.0046 (n=6+13); i. pvalue<0.0887 (n=15+14); j. pvalue<0.5506 (n=15+11)

**Table A_4b: Efficiency gains – Panel data regressions**

|  | (1) | (2) | (3) | (4) |
| --- | --- | --- | --- | --- |
| Efficiency gains | VCM - NP | VCM - Pun | WLM - NP | WLM - Pun |
| Period | -0.424*** | 0.517 | 0.797* | 0.472* |
|  | (0.118) | (0.393) | (0.443) | (0.251) |
| Symmetric profile | -7.584 | 8.835 | 20.93*** | 38.06*** |
|  | (6.330) | (8.481) | (5.784) | (7.780) |
| Constant | 25.38*** | 2.535 | 6.872 | 2.866 |
|  | (1.907) | (5.650) | (4.293) | (8.050) |
| Observations | 1,200 | 1,200 | 1,120 | 1,120 |
| Number of subjects | 60 | 60 | 56 | 56 |

Robust standard errors in parentheses

*** p<0.01, ** p<0.05, * p<0.1

Table A_4a presents some descriptive statistics of efficiency gains, as described in the text, across games and conditions and dividing the whole sample in symmetric and asymmetric contribution profiles. Following the analysis of effort choices, the non-parametric analysis shows that the effect of punishment is again heavily mediated by the game: efficiency gains are substantial in both WLM symmetric profiles (with and without punishment), and asymmetric profiles obtain similar results in both conditions across both games, as no differences are observed between asymmetric profiles with punishment, and gains are only significantly different at a marginal level without punishment. Again, symmetric profiles are rare in the VCM, so Table A_4b presents the outcome of panel data regressions showing similar results to the ones obtained in the analysis of effort choices.

**Figure A_5: Effort and efficiency gains across conditions**

Figure A_5 presents the evolution of effort and efficiency gains across the 20 rounds of the game, without punishment (top figure; first block of 20 rounds) and with punishment (bottom figure; second block of 20 rounds). A simple way of extracting information from these figures is to observe the distance between efficiency gains and effort choices in both blocks. Without punishment, this distance measures the cost of miscoordination in the WLM; no distance is observed in the VCM because every contribution generates a group benefit and miscoordination is impossible. When sanctions are available, the distance between efficiency gains and effort choices capture both miscoordination any punishment costs. So, in the bottom figure, efficiency gains are always below the average effort decision. Note that this analysis is possible because the scale of effort choices (from 0 to 50, the round endowment) and the scale of efficiency gains (from 0, as in the no contribution profile, to 50, as in the case in which every group member fully contributes to the public good) coincide.

It is easy to capture the differences between games and condition by looking at the second half of the rounds. When this second half starts, in round 11, efficiency gains are around 30 (60% of the maximum) in the WLM both without and with punishment (31.11 and 31.38, respectively; the difference is not significant, according to a Wilcoxon signed rank test, pvalue<0.1707). In the last ten rounds of each block, efficiency gains follow very different patterns in the WLM. While they sky rocket to 46.38 in round 19 (92.74% of the maximum) with sanctions, they keep remarkably flat without punishment (30.44, or 60.8% of the maximum, in the same round 19). The distance between average effort and average efficiency gains vanishes when sanctions are available, suggesting that groups coordinate extremely well, without using a significant amount of punishment.

In the VCM, however, the picture is very different. Even when punishment is the only cost subjects incur in the second block of 20 rounds, the distance between effort and efficiency gains are substantial and do not diminish over time. While effort is around 60% of the maximum in round 11 of the second block, when punishment is available (30.40, or 60.80% of the maximum), efficiency gains stay low, around 20% of the maximum (or 10 units above the no-contribution profit). This substantial distance is consistent with the much higher frequency and intensity of punishment documented in the main text.

**Table A_6a: Total punishment sent – Descriptive statistics and non-parametric tests**

|  | Asymmetric | Symmetric |
| --- | --- | --- |
| VCM | 2.77 | 1.37 |
|  | (4.28) | (3.66) |
| WLM | 1.34 | 0.24 |
|  | (3.14) | (2.00) |

**Figure A_6b: Total punishment sent – Distribution by game and condition**

**Table A_6c: Total punishment sent – Panel data Tobit regressions**

|  | (1) | (2) |
| --- | --- | --- |
| Total punishment sent | VCM | WLM |
| Period | -0.108*** | -0.0393 |
|  | (0.0313) | (0.0971) |
| Symmetric profile | -3.296*** | -8.489*** |
|  | (0.785) | (1.497) |
| Constant | 1.438** | -8.592*** |
|  | (0.675) | (1.652) |
| Observations | 1,200 | 1,120 |
| Number of subjects | 60 | 56 |

Standard errors in parentheses

*** p<0.01, ** p<0.05, * p<0.1

Table A_6a presents the descriptive statistics of the total number of points sent by participants in the two games, across symmetric and asymmetric effort profiles. As supported by the left censored Tobit regressions in Table A_6c, punishment is substantial and significantly smaller when participants make the same decision in both games. Note that when contributions to the public good are aligned, there is no room for social or antisocial punishment, as all individuals contribute the same. Bur, being a repeated game, there is still a rationale for sanctions linked with previous interactions (e.g. through blind retaliation).

Figure A_6b presents the distribution of the total punishment points sent across the four cases considered in Table A_6a. The proportion of participants not punishing at all in the symmetric cases range from slightly below 80% in the VCM to around 95% in the WLM. When decisions differ, as in the asymmetric cases, more (slightly less) than half of the participants punish in the VCM (WLM).

**Table A_7. The intensity of sanctions as a function of different reference thresholds**

| Panel data Tobit  (left censored) | (1) | (2) |
| --- | --- | --- |
| WLM | -5.837*** | -6.164*** |
|  | (0.886) | (0.874) |
| Period | -0.0567*** | -0.0700*** |
|  | (0.0147) | (0.0161) |
| Period*WLM | 0.0183 | -0.0192 |
|  | (0.0343) | (0.0380) |
| Above target | 0.159*** |  |
|  | (0.00777) |  |
| Above average |  | 0.0771*** |
|  |  | (0.0133) |
| Above target*WLM | 0.137*** |  |
|  | (0.0236) |  |
| Above average*WLM |  | 0.206*** |
|  |  | (0.0437) |
| Below target | 0.00531 |  |
|  | (0.00868) |  |
| Below average |  | 0.00341 |
|  |  | (0.0120) |
| Below target*WLM | -0.0106 |  |
|  | (0.0294) |  |
| Below average*WLM |  | -0.0237 |
|  |  | (0.0377) |
| Constant | -3.236*** | -2.545*** |
|  | (0.532) | (0.526) |
| # Obs. | 6,960 | 6,960 |
| # Subjects | 116 | 116 |
| Log likelihood | -5223.6268 | -4933.4247 |

Standard errors in parentheses

*** p<0.01, ** p<0.05, * p<0.1

Table A_7 presents the results of estimating two left censored Tobit models with random effects at the individual level and clustering at the group level. Model 1 in the table uses the average contribution to the public good as the threshold to determine whether punishment is social or antisocial, while Model 2 uses the contribution of the punished individual (following the different concepts used in the literature, see Bochet et al., 2006, Cinyabuguma et al., 2006, and Herrmann et al., 2008). The dependent variable is the number of punishment points sent, and the independent variables are a game dummy (WLM), a time trend (Period), the interaction term between period and game (Period*WLM), and the distance between the threshold contribution (group average or punisher’s contribution, respectively) and the contribution of the individual punished.

We introduce different independent variables to analyse antisocial (when the contribution of the punisher is below the threshold) and social punishment (when the contribution of the punisher is above the reference threshold): Above average and Above target (Bellow average and Bellow target). We also include interaction terms to control for different behavioral patterns in the VCM and the WLM.

Note that these variables account for the absolute distance between the contribution of the punisher and the reference threshold. These variables explain how the intensity of the deviation with respect to the norm (the reference threshold) triggers the amount of punishment points sent. By design, this analysis does not address frequency of punishment; it rather specifically addresses how punishment intensity depends to the distance to the norm (using the two reference thresholds as proxies).

The results show that punishments significantly decreases in the WLM, that social punishment significantly depends on the distance to the norm (and significantly more in the WLM than in the VCM), that the distance to the norm does not explain the intensity of antisocial punishment in either games, and that both reference thresholds generate very similar results.
